# 

**Published:** 1848-02

**Authors:** 


### INDEX.

Abdominal Tumor, Extirpation of 193
Abstract of Med. Science, 254, 521
Aborigines, Phys. condition of 310
Addresses, 70
Adulterations of Medicines, 350
Air Passages, For. Bodies in 121
Albany Medical Dinner, 83
Allaire's cases of Foreign Bodies in the Air Passages, 121
Alcohol, test of the Purity of 361
American Medical Almanac, 422
Amer. Jour. of Med. Science, 255
Antagonism between Fevers, 544
Aneurismal Varix, case of 505
Anatomy, Hand-Book of 251
Army, Medical Staff of 451
Armor on Fevers, 198
Armor's Letter, 490
A Prize, 189
Asylums, Lunatic 39
Asylum, Deaf and Dumb 431

Benton on the use of Strychnia in Fevers, 299
Bicknell's case of Glossitis, 305
Bird's Dispensary Report, 504
Blaney on Chloroform, 561
Blisters, effects of, on Young Subjects, 266
Blood-letting, effects of, on Young Subjects, 441
Bloodletting, a new criterion for 523
Books, Medical 59, 92
Botany, Medical work on 252
Brainard, on injection of Tr. Iodine in Spina Bifida, 475
Brainard on Strychnia in Intermittent Fever, 114
Brainard on astringent injections in Profuse Suppuration, 418
Brainard's case of Extirpation of Abdominal Tumor, 193
Burns treated with Ammonia, 360
Brainard's case of Aneurismal Varix, 505

Camphor, poisoning by 167
Carson's Medical Botany, 430
Cervix Uteri, Elongation of 73
Chest, Diseases of 46
Chelius' System of Surgery, 428
Children, case of four at a birth, 452
Children, Treatise on Diseases of 251
Chicago Hospital, 85
Chicago Dispensary, 504
Chloroform, 457, 458, 459, 480, 561
Chinese Physiology, 80
Cholera, 452
Clark on Strychnia in Bronchitis, 12
Clinical Instruc. at Chicago, 171
Clippinger's Letter on Sulph. Ether, 130
Clinical Lectures, 17, 74, 126
Cold Water in Fever, 528
College, Rush Medical 90
Columbus Medical College, 90, 282
*Concours*, Election by 59
*Concours*, the system of 378
Congress, memorial to 453
Convention, National Medical 179
Copland's Medical Dictionary 169
Cotton in Linen, means of testing for 362
Croup, Nitrate Silver in 551
Croup, Treatment of 369
Croup, Gastric origin of 374

Davis' case of Rupture of the Uterus, 222
Dental Register of the West, 422
Disease as modified by marching and a camp life, 225
Deaf and Dumb Asylum, 431

Electricity, influence of, on the Human System, 357
Encyclopedia Americana, 233
Ether Sulph., 130, 167, 168, 382, 411, 537
Etherization in Midwifery, 293
Etherization, 263
Ether, resuscitation from effects of 376
Evans, on Prolapsus Uteri, and its treatment, 103
Eye-Ball, Excrescence on 16
Eye, case of injury of 263

Fever, Report on 1
Fever, Intermittent and Remittent 48
Fever, Pathology and treat. of 385
Fitch on Radical cure of Inguinal Hernia, 102
Fitch's Clinique on Chron. Peritonitis, 126
Fitch on Infla. of the Brain, 212
Fownes Elementary Chemistry noticed, 348

Galvanism in Uterine Hemorrhage, 470
Gardner's Med. Dictionary, 436
Generation, Treatise on 423
Geological Survey, 188
Glossitis, a case of 305
Golliday's Critique on Henry's treatment of Fevers, 218
Green's treatise on Diseases of the Air Passages, 437

Heart, Fibrous Concretion in, by G. Sprague, M. D., 402
Heart, Diseases of 133
Henry on Pathology and Treatment of Fever, 385
Hernia, Inguinal, radical cure of 102
Herrick's case of Diseased Kidneys, 307
Herrick on Disease as modified by marching and camp life, 225
Herrick on Surgery at Buena Vista, 300, 414
Horr's case of Etherization, 411
Hospital, Chicago 85
Human Tripod, 261
Hydrocephalus, Hydroid. Pottass in 97, 279

Illinois Insane Hospital, 89
Indiana Union Med. Society, 480
Inflammation of the Brain, 212
Insane Hospitals, 39, 158, 522
Insane Hospital, Chicago 280
Insanity, Hereditary transmission of 359
Insane Clergyman, anecdote of 377
Intermittent Disease, various forms of 407
Introductory Lectures, 478
Intestinal Mucous Memberane, Path. of, in Infancy, (trsl.) 495
Iowa Farmer's Advocate, 480

Jones on Salivation by Chemical Action, 409

Kidneys, case of Disease of 306

Labor, Remarkable case of 492
Lactation, Protracted case of 62
Legislature of Conn., 258
Letter from Dr. Armor, 490
Letheon, 76, 83, 87, 189
Lightning, accidents by 280
Lisfranc, Death of 169
Lunatic Asylums, 39, 84

Malaria, its source, in the South and West, 540
Martico, 61
Martin on Intermittent Disease, 407
McGirr on Etheri. in Mid., 293
McGirr on Strychnia in Intermittent Fever, 481
McLean on Tannate of Quinia 289
McNutt on Milk Sickness, 205
Mead on Hydro. Pot. in Hydrocephalus, 97
Med. Society, Indianapolis 561
Med. Science, Bulletin of 92
Medical Societies, 174
Medical Horoism, 374
Medical Legislation, 176
Medical Schools, vacancies in 186
Medico-Chirugical Review, 258
Medical Profession, Organization of 276
Medical Profession, 284
Meek on Physical condition of the Aborigines, 310
Mercury, effects of, on Young Subjects, 160
Milk Sickness, Observations on 205
Miscellaneous Intelligence, 285
Morphia Muriate in Toothache and Neuralgia, 376

Nitrous Oxide, Inhalation of 423
North-Western Educator, 423

Obituary Notices, 93, 190 480
Obstetrics, New work on 283
Ohio Medical College, 377
Opthalmia, Inhalation of Ether in 537
Opthalmia of Infants, Douches in 538
Our Journal 560
Ovariotomy, case of 259

Pain, mitigation of, by Narcotic Vapors, 166
Palate, Instrument for 67
Parturition, Ether in 168
Parturition, natural & difficult 26
Periodical Fevers, Pathol. and Treatment of 198
Pertussis, Alum in 356
Philadelphia Col. of Med. 91
Physicians, Fate of 375
Piper Angustifolium, 61
Placenta, Artificial separation of 261
Preg., Tumor mistaken for 554
Pregnancy, Menstruation during 547
Prince on Fever, 1
Prolapsus Uteri, and Treat., 103
Providence Insane Hospital 84
Professional Benevolence, Public Claims on 257

Quackery and the Newspaper Press, 263
Quackery by Apothecaries, 357
Quackery, Illustrations of 362
Quackery, Antidote for 472
Quinia, Tannate of 289
Quinine, on the use of 264
Quinine in Coffee, 262, 361
Quinine, Arseniate of 70

Rankin's Abstract, 254, 521
Rattlesnake, Bite of 549
Rape under the Influence of Ether, 376
Reading Room of Rush Med. College, 279
Reese's Analy. of Physiology, 348
Request, 132
Rheumatism of the Heart, 123
Rock River Med. Society, 412
Rush Medical College, Clinical Lectures in 17
Rush Medical College, 90, 282
Rush Medical College and the National Convention, 468

St. Louis Med. and Surg. Jour. 282
Salivation by Chem. Action, 409
Selling a Practice, 281
Sex, Doubtful case of 64
Spina Bifida, Cure of, by Tinct. Iodine, 475
Sprague's case of Fibrin. Concretions in the Heart, 402
Sprains, Treatment of 65
Spontaneous Evolution of Fœtus, 260
Splints, 553
Strychnia in Bronchitis, 12
Statistics of Med. Schools, 91
Strychnia in Fevers, 114, 299, 481
Still Born Children, 546
Stahl's translation of Remarks on Path. Mucous Mem., 495
Suppuration Profuse, Use of, Astringent Injections in 418
Surgery at Buena Vista, 300, 414
Surgery Operation, New Elements of 46

University of Pennsylvania, 377
Urine, Retention of, in Labor, 81
Urine Alkaline, in Health, 545
Uterus, case of Rupture of 222
Uterine Disease, a Prac. treat. on 254, 319

Vade Mecum for Students, 252
Vacant Chairs in Med Schools, 187

Wadsworth's case of Metastasis in Rheumatism, 123
Water, use of, in Fever, 528
Warren, Dr. Resignation of 92
Watson's Lectures, 435
Welch's Letter, 492
Whitman, Mr. Case of 548
Wisconsin Med. Society, 553
Wood's Treatise on the Prac. of Medicine, 338
Womb, Case of Rupture of 539
Wounds, New Method for the Union of 359

### TO READERS AND CORRESPONDENTS.

We have received since the publication of our last number—

A communication from our much esteemed correspondent, Dr. Stahl, of Quincy. Also, a communication from Dr. Mathews—both of which will appear in our next.

The Principles and Practice of Midwifery. By David H. Tucker, M. D., &c. It being No. 1 of The Medical Practitioners' and Students' Library. From the Publishers, Lindsey & Blackiston.

Lecture on Statistical Medicine. By Samuel A. Cartwright, M.D.

Also, numerous Introductory Lectures to Courses in the different Medical Schools, and Catalogues

Of the University of Pennsylvania.
Of the Pennsylvania College of Medicine.
Of the Medical Institute of Philadelphia.
Of the Medical Department of New York University.
Of the College of Physicians and Surgeons, N. Y.
Of the Medical Department of the University of Louisville.
Of the Medical Department of Transylvania University.
Of the Medical Department of St. Louis University.
Of the Medical College of Georgia.
Of the Sterling Medical College, and
Of the Medical Department of Illinois College.

### IN EXCHANGE.

The Boston Medical and Surgical Journal to March.
The Medical Examiner to March.
The New York Journal of Medicine to February.
The British and Foreign Med. Chir. Review, for January, 1848.
The Medical News and Library to March.
The Buffalo Medical Journal to March.
The Western Lancet to March.
The Western Journal of Medicine and Surgery to March.
The Southern Medical and Surgical Journal to February.
The New Orleans Medical and Surgical Journal to March.
The St. Louis Medical and Surgical Journal to February.
The Missouri Medical and Surgical Journal to March.
The American Journal of Insanity for January, 1848.
The Journal of Health and Prac. Educator for March.
The Dental Register of the West. Vol. 1. No. 1.

---

### LIST OF PAYMENTS FOR FEBRUARY AND MARCH.

| | | | |
|---|---|---|---|
| Dr. A. G. Henry, | $2 | Dr. T. S. Mills, | $4 |
| " Bassett & Snyder, | 2 | " M. T. Irwin, | 2 |
| " John Stickel, | 2 | " J. Bennett, | 2 |
| " James Adair, | 2 | " J. S. Baxter, | 1 |
| " D. D. Wait, | 4 | " Z. Doherty, | 2 |
| " T. Judd, | 1 | " N. Woodyard, | 1 |
| " A. B. Price, | 2 | " Miller & Durr, | 3 |
| " E. Webster, | 2 | " J. Beecher, | 2 |
| " Saml. Elder, | 3 | " W. H. Prestley, | 4 |
| " E. Cooper, | 2 | | |

### PROSPECTUS

OF THE

### NORTH-WESTERN MEDICAL & SURGICAL JOURNAL.

THE ILLS. AND IND. MED. AND SURG. JOURNAL will be published hereafter under the title of THE NORTH-WESTERN MEDICAL AND SURGICAL JOURNAL, and will continue to be issued simultaneously at Chicago, Ills., and Indianapolis, Ind., on the first of every other month, beginning with May, in numbers, each containing from 88 to 96 pages, neatly printed with new type and on good paper, and which at the end of the year will constitute a volume of about 550 pages octavo, with title page and index complete.

Contributions, especially from Physicians of the North-West, are earnestly solicited.

TERMS.—Two DOLLARS per annum, *in advance*, or Eight Dollars in one remittance for Five Copies, which may be sent by mail at our risk, or paid to either of the Editors or following authorized Agents: Dr. J. B. Herrick, Vandalia, Ills.; Dr. Sample Loftin, Mr. J. M. Phipps, Ind., or Mr. Orrin T. Maxson, W. T.

W. B. HERRICK, M. D., Chicago,
JOHN EVANS, M. D., Indiana,
*Profs. in Rush Med. College—Eds. and Proprietors.*

